# The views of family physicians on National Health Insurance in Gauteng Province, South Africa

**DOI:** 10.4102/safp.v66i1.5831

**Published:** 2024-03-25

**Authors:** Shane D. Murphy, Klaus von Pressentin, Shabir A. Moosa

**Affiliations:** 1 Department of Family Medicine, University of the Witwatersrand, Johannesburg, South Africa; 2 Division of Family Medicine, Department of Family, Community and Emergency Care, University of Cape Town, South Africa

**Keywords:** family physician, NHI, National Health Insurance, Universal Health Coverage, UHC, District Health System, DHS, governance

## Abstract

**Background:**

Universal health coverage (UHC) improves national health outcomes while addressing social inequalities in access to quality healthcare services. The district health system (DHS) is critical to the success of UHC in South Africa through the National Health Insurance (NHI) scheme. Family physicians (FPs), as champions of primary care, are central to the DHS operation and implementation of NHI.

**Methods:**

This was a qualitative exploratory study that used semi-structured interviews to explore FPs views and engagement on NHI policy and implementation in their districts. Ten FPs were included through purposive sampling.

**Results:**

Most of the FPs interviewed were not engaged in either policy formulation or strategic planning. The NHI bill was seen as a theoretical ideology that lacked any clear plan. Family physicians expressed several concerns around corruption in governmental structures that could play out in NHI implementation. Family physicians felt unsupported within their district structures and disempowered to engage in rollout strategies. The FPs were able to provide useful solutions to health system challenges because of the design of their training programmes, as well as their experience at the primary care level.

**Conclusion:**

Healthcare governance in South Africa remains located in national and provincial structures. Devolution of governance to the DHS is required if NHI implementation is to succeed. The FPs need to be engaged in NHI strategies, to translate plans into actionable objectives at the primary care level.

**Contribution:**

This study highlights the need to involve FPs as key actors in implementing NHI strategies at a decentralised DHS governance level.

## Introduction

Globally, countries are implementing comprehensive healthcare reforms through Universal Health Coverage (UHC) to drive equity in access to quality health services. Universal Health Coverage is posited by the World Health Organization (WHO) as a means for governments to improve national health levels while cross-subsidising population risk and enhancing social equity.^1,2^ Central to the ideology of UHC is an effective primary healthcare (PHC) service, recently cited by the Lancet Global Health Commission on financing PHC as a ‘key component of all high-performing health systems’ and ‘an essential foundation’ of UHC.^3^ In developing countries such as South Africa, a transformation from the current hospital-centred model of healthcare delivery to a decentralised model that prioritises PHC services is needed to successfully implement NHI.

**FIGURE 1:**
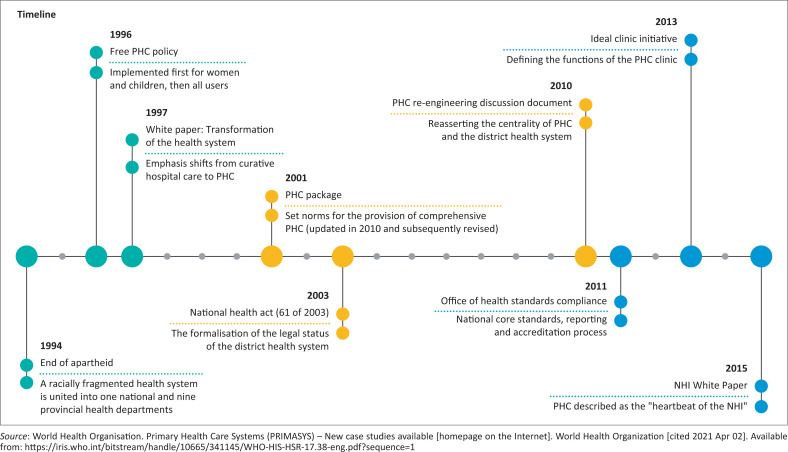
Primary health care strategies since 1994.

The South African government began the phased implementation of a national health insurance (NHI) scheme to drive the implementation of UHC in 2011.^4^ National Health Insurance is a health-financing scheme that is intended to pool funds and facilitate cost-effective and accountable purchasing of healthcare services that is responsive to population needs. Coupled with the financial reforms of the NHI White Paper as well as the NHI Bill, the documents underscore the importance of PHC strengthening and spell out governmental commitment to PHC strengthening. This commitment, however, is not new to the South African setting, as the government has embedded PHC strengthening at the heart of health sector reform since the advent of democracy in 1994 (Figure 1).

**FIGURE 2:**
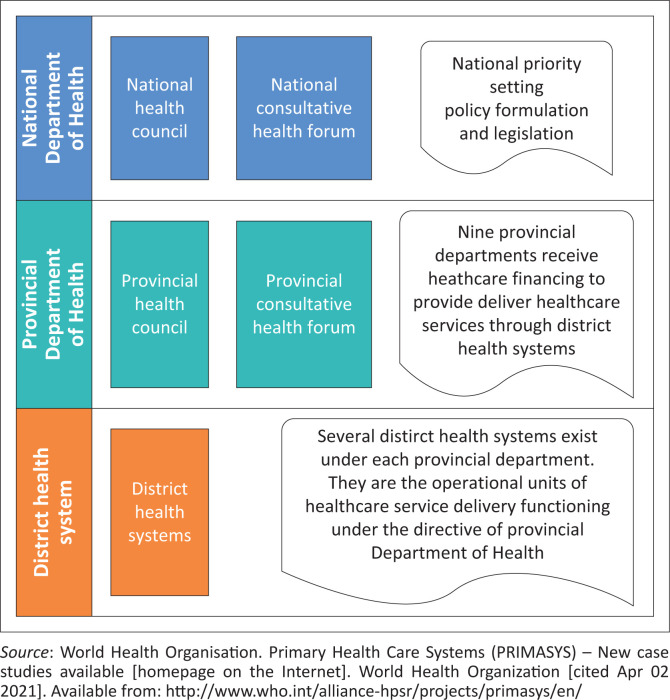
Healthcare framework model.

**FIGURE 3:**
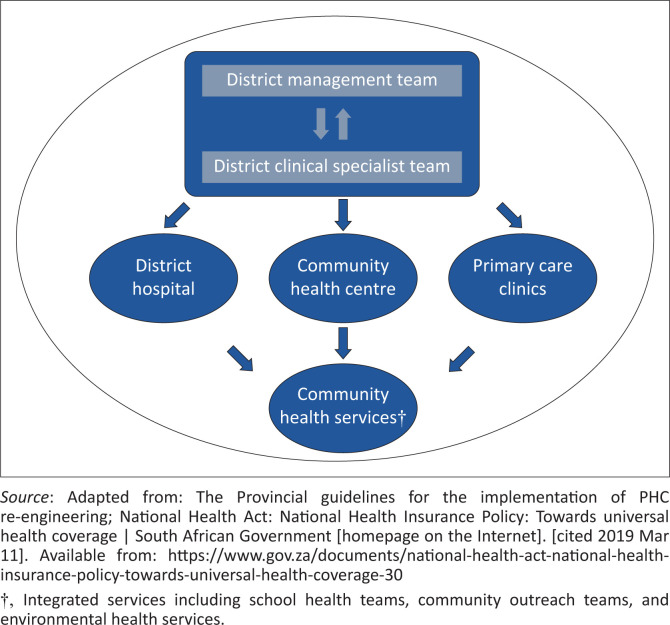
Structure of the district health system.

The South African healthcare framework, as legislated by the *National Health Act* of 2003, is a top-down model consisting of three tiers (Figure 2): the National Department of Health (NDOH), provincial departments of health, and district health systems (DHSs). The NDOH oversees policy formulation and national priority setting, while provincial departments, receiving the bulk of healthcare financing, are responsible for healthcare service delivery through the DHS as an agent of the province. The DHS (see Figure 3) is a functional unit of provincial departments responsible for healthcare service delivery to health districts (defined as ‘a well-defined population, living within a clearly delineated administrative and geographical area’). Funding, priority setting, and decision-making around healthcare services are conducted by provincial departments and prescribed to health districts.

A critical component of the NHI Bill is the re-structuring of the extant centralised (see Figure 1) healthcare model to a decentralised model where governance duties and responsibilities for PHC strengthening are devolved to the DHS. The establishment of District Health Management Offices (DHMOs) is intended to decentralise healthcare decision-making from the province to the health district to increase transparency and accountability, as well as foster contextually appropriate healthcare service delivery. The DHS is pivotal to primary care strengthening^5^ and its success is a strong determinant of UHC implementation success.^6,7,8^ In South Africa, each health district consists of a management structure that governs the operations of facilities within the district, including district hospital(s), community healthcare centre(s), and primary care clinics.^1,9^ These facilities further liaise and collaborate with community-based services to enhance population coverage and access to care.

The district management structure oversees the delivery of healthcare services within the respective district and consists of a district management team (DMT) and a District Clinical Specialist Team (DCST). These two teams are intended to work interdependently to oversee healthcare services within their respective district. The DMT includes non-clinical functions such as human resources, procurement, pharmacy, and ancillary services, while the DCST consists of a team of four medical specialists: a family physician (FP), an obstetrician and gynaecologist, a paediatrician, and a general surgeon. The medical specialties represented within the DCST target the national disease profile. The FP is intended to oversee the DCST as a primary care specialist and facilitate integrated team-based primary care.^10^

Family physicians are primary care specialists who have completed a 4-year postgraduate training programme in the discipline of family medicine. Comparatively, most of the generalist doctors in SA enter practice as registered medical practitioners after undergraduate training alone. The distinct professional identity of FPs as agents of change, unique in their primary care expertise and a systems-oriented lens facilitates multidisciplinary collaboration, referrals and gate-keeping stewardship, and health system governance both internationally^11^ as well as in SA.^10,12,13^ Furthermore, the family medicine curriculum is situated in PHC and DHS services, whereas other medical specialists are trained in central tertiary hospitals.^13^ This contrast in training context allows FPs’ unique insight into the operationalisation of NHI through PHC and DHS services.^14^ The value of the FP in the DHS and PHC can be linked to the roles embedded in the family medicine curriculum (Table 1).

**TABLE 1:** Six roles of the family physician in the district health system.

Role	Description
Care provider	A competent clinician, able to deal with most of the health problems in the community that he or she serves, within the district hospital or primary care setting, and competent in relational skills (including patient centeredness, communication skills, and bio-psychosocial approach).
Consultant	As part of a well-functioning healthcare team, he or she provides support to other practitioners (e.g. clinical nurse practitioners and medical officers) by seeing referred patients in primary care facilities or district hospitals.
Capacity builder	As a senior clinician, he or she is responsible for the mentoring and training of less qualified clinicians within the primary health care or district hospital teams.
Clinical trainer	He or she may need to function as the clinical supervisor in the workplace for medical students, clinical associate trainees, interns, or registrars.
Clinical governance leader	Responsible for improving the quality of clinical services within the sub-district and facility where he or she is appointed.
Champion of community-orientated primary care (COPC)	Supports the development of a COPC approach to the district health system, particularly the development and integration of ward-based outreach teams (a team of community health workers responsible for a geographically defined group of households).

*Source*: Von Pressentin KB, Mash RJ, Baldwin-Ragaven L, et al. The perceived impact of family physicians on the district health system in South Africa: A cross-sectional survey. BMC Fam Pract. 2018;19(1):24. https://doi.org/10.1186/s12875-018-0710-0

## Implementation of family medicine in Gauteng

Despite the compelling evidence for the utility of FPs in improving the DHS and PHC, as well as the disciplines sustained lobbying and engagement in health systems strengthening, their role remains under-appreciated. Inclusion in recent policies such as the National Development Plan, Ideal Clinic Standards policy, and the Human Resources for Health (HRH) Strategic Plan are limited, and provide only vague descriptions of roles and responsibilities.^15,16^ The HRH strategy emphasises the role of FPs in improving the quality and coverage of PHC. Furthermore, the strategy provides for DCSTs as a cross-disciplinary approach to enhance clinical governance and healthcare systems strengthening within PHC.^14,17^ Despite this, FPs remain in absolute shortfall^12,17^ and, as the champions of PHC, they do not enjoy the support and authority to engage with their intended purposes.

Several local studies have sought to explore the awareness and views of primary care healthcare practitioners on NHI.^18,19,20,21,22,23,24^ However, most studies have looked at private sector general practitioners only, finding common themes – HCPs supported the ideology of NHI, but distrust towards government, poor communication and engagement, governmental capacity to improve primary care, and ambiguity on what the implementation strategy was, or what the implications would be for HCPs in primary care.^18,19,20,21,22,23,24^ Studies of hospital-based staff cited primary concerns around the state of the public sector. Respondents felt that the need to improve service delivery in the primary care setting was a critical starting point before any other plans should be considered.

Research on non-clinical staff (district and sub-district managers) similarly found poor awareness and engagement on NHI with limited experience of the proposed devolution of governance to the district level.^25^ Research on the role of family medicine in NHI showed that primary care practitioners (non-FPs) strongly support the pillars of family medicine, and view the discipline as a linking mechanism to improve primary care and ensure clinical governance and oversight.^10^ The impact made by FPs in DHS facilities across seven South African provinces was perceived by co-workers as high for five of the six agreed roles: care provider, consultant, clinical trainer, leader of clinical governance, and champion of community-orientated primary care. Their impact in the role of capacity builder was significantly less than the other roles although still seen as a moderate impact.^26^

Despite the evidence that specialist FPs are critical to PHC re-engineering, no national policy documents include them, and no research has yet reviewed their views and engagement with NHI. The aim of this article was to explore the awareness and views of FPs in Gauteng Province on NHI and their role in its implementation.

## Methods

### Study design

This was a phenomenological exploratory qualitative study founded on interpretivism.

### Study setting

Our study was conducted across four health districts within Gauteng Province, South Africa between March 2020 and September 2020. The respective districts were selected as they have established family medicine centres linked to tertiary education training programmes for family medicine.

### Study population and sampling

Sampling was conducted through a framework of stratified random sampling so that a balanced mix of FPs from all backgrounds would be sampled. All participants had completed their specialist training in family medicine, were employed in the public sector, and affiliated with a family medicine training programme. Purposive sampling was then used to identify data rich respondents within this sampling framework.

### Data-collection process

Semi-structured interviews were used for a non-directional and detailed exploration of respondents’ perspectives. Email invitations, which included a participant information sheet, were sent to prospective participants. A protocol of two reminders, each sent 1 week apart, was used to remind non-responders. There were no non-responders throughout the sampling process. Because of the coronavirus disease (COVID-19) pandemic, interviews were conducted with consenting participants by encrypted telecommunication software, as regulated by the local ethics committee. The following three core questions were included, based on the findings from the literature review and the research objectives, and were agreed upon by the researchers:

1. What are your views on NHI?
2. Can you tell me about your engagement in NHI policy development?
3. What is your perception of the implementation of NHI?

A final non-directional question (‘Is there any other opinion or thought that you would like to express?’) was used to facilitate idiosyncratic thematic emergence.

Interviews were recorded and transcribed verbatim. The concepts of ‘information power’ and ‘informational redundancy’^25^ informed pragmatic sampling saturation. The concepts of ‘information power’ and ‘informational redundancy’^27^ informed pragmatic sampling saturation. Transcripts were sent back to participants for member-checking. No changes were made following member-checks.

### Data analysis

Transcripts were imported into computer-assisted qualitative data analysis software^28^ to facilitate robust and explicit data analysis. Data analysis followed the five-step deductive approach of the Framework Analysis^29^: familiarisation, framework formulation, coding and indexing, charting, and mapping and interpretation. Peer-checking of the thematic analysis, as well as the greater framework analysis, was performed by the second researcher. No divergent interpretations arose during this process. Measures to ensure the trustworthiness of this work are listed in Table 2.

**TABLE 2:** Ensuring trustworthiness.

Component of trustworthiness	Measures
Credibility	This study used the validated Framework Analysis method. Critical reflexivity was used during interviews with thick descriptions of data to facilitate internal validity.
Transferability	Detail of the research setting with an audit trail is provided for future research and cross comparison.
Confirmability	Computer-assisted qualitative data analysis software was used to leave a paper trail alongside reflexive commentary. Quotes are used for in vivo description.
Dependability	Study design and methods are made explicit so that researchers can emulate the study design.

*Source*: Murphy SD, Moosa S. The views of public service managers on the implementation of National Health Insurance in primary care: A case of Johannesburg Health District, Gauteng Province, Republic of South Africa. BMC Health Serv Res. 2021;21(1):969. https://doi.org/10.1186/s12913-021-06990-4

### Reflexivity

The principal investigator, with formal training on how to conduct interviews and previous works of the like, SM, conducted all of the interviews. The principal investigator worked in the same district as three of the study participants. While every effort was made to maintain objectivity during the interview process as well as the data analysis, it is possible that bias (in particular, social desirability) may have been introduced. Parallel data analysis by the senior researchers helped to mitigate this bias in the interviews. The senior researchers, S.A.M. and K.v.P., have extensive experience in qualitative research. All researchers are FPs jointly appointed by the Department of Health and an academic institute.

### Ethical considerations

Ethics committee approval of the study was obtained from the University of Witwatersrand’s Human Research Ethics Committee (M191046), as well as the Research Committee of the Johannesburg Health District (GP_202006_048).

## Results

Ten participants were interviewed in this study. Sampling saturation was reached after seven interviews, while a further three were conducted which revealed no deviant or novel information. All FPs were based in urban districts around Gauteng province, South Africa. A total of four health districts were represented: Tshwane, Sedibeng, Ekurhuleni, and the Johannesburg Metropolitan area. All participants were affiliated with a local university. Of these, two were academic appointees, while the rest were joint appointees (variable split of employment between the public health sector and a medical university). Only one respondent had less than 10 years of experience, having recently assumed the role of head of the district clinical services. The respondents had varying levels of familiarity with the NHI policy and roll out strategies. Table 3 describes the distribution of participants. The four overarching themes that were observed included: views on NHI, communication and engagement, obstacles to implementation, and suggestions for success.

**TABLE 3:** Description of respondents.

Respondent	District	Gender	Experience in current setting	Work setting
1	Metro†	Male	27 years	Clinic
2	Ekurhuleni	Male	19 years	Clinic and hospital
3	Tshwane	Male	12 years	Clinic
4	Metro†	Female	23 years	Clinic
5	Ekurhuleni	Female	22 years	Clinic and hospital
6	Tshwane	Male	24 years	Hospital
7	Sedibeng	Male	6 months	Hospital
8	Metro†	Female	12 years	Clinic
9	Tshwane	Male	34 years	Clinic
10	Sedibeng	Male	5 years	Clinic

*Source:* World Health Organisation. Primary Health Care Systems (PRIMASYS) – New case studies available [homepage on the Internet]. World Health Organization [cited 2021 Apr 02]. Available from: http://www.who.int/alliance-hpsr/projects/primasys/en/

† , Metro: Johannesburg Metropolitan area.

## Views on National Health Insurance

### Ideological alignment

The FPs in our study uniformly viewed UHC as a moral obligation. Furthermore, FPs supported the implementation of NHI for its potential to provide redress to current social inequities and the social determinants of health:

‘[*L*]ook, it’s a lovely idea … It’s going to actually balance the inequality that is in the healthcare in South Africa.’ (R7, Male, Sedibeng)

‘I think that it comes from a, greater good, perspective. I think there needs to be change in our current system, because it only, obviously, works for the privileged few.’ (R8, Female, Metropolitan)

### Hesitations around practical implications

The support of the ideology within NHI seemed to fade as the discussions moved towards translating the conceptual framework to meaningful change in healthcare service delivery. Concerns were raised around the NHI bill’s focus on financing mechanisms, without much description of any real strategy:

‘Ja, I think it’s a great idea, ja, but like with a lot of things, the devil is in the details.’ (R3, Male, Tshwane)

‘The official documents, if you look at it, is about a funding model; is about the funding model, and the funding mechanisms, with no real intention of looking at healthcare.’ (R9, Male, Tshwane)

### Distrust towards current government and health authorities

Distrust towards the current governing structures was a major feature of FP views on the success of NHI. The basis of this sentiment was ascribed to FP perceptions of how other state-owned enterprises (SOEs) were unsuccessful and subject to high levels of corruption:

‘And then my other comment is, I don’t think it’s about throwing money at the collapsed system, to resurrect it, it will just fuel corruption … Because why? Because money comes into it, and a lot of money; then you’re going to have unethical behaviour, even from professionals, because somebody will try to cheat the system and get as much money as possible, meanwhile not actually providing the service.’ (R1, Male, Metropolitan)

‘I think there’s so much suspicion in this country, because it comes down to the intention; intentions of people. And I think there’s been a real loss of trust in society, and rightly so, because of the way we see things get handled. I think things need to change, but I also don’t trust the government system to do a good job.’ (R8, Female, Metropolitan)

‘But it’s actually sadness. It is the same type of sadness that you would get when you look at SARS [*South African Revenue Service*] and ESKOM [*Electrical Supply Commission*], or maybe the most important one is PRASA [*Passenger Rail Association of South Africa*]; you are sad for what could have been.’ (R9, Male, Tshwane)

## Communication and engagement

### Low levels of engagement

Family physicians reported mixed levels of communication from the government with regard to opportunities for input into policy formulation and NHI roll out, commonly stating that their awareness of NHI was informed by other sources:

‘No, no, no, only these lectures that I have attended, but it was a matter of interest.’ (R4 Female, Metropolitan)

‘I mean, it’s often just the sort of the media discussion.’ (R3, Male, Tshwane)

In contrast to this, FPs from one district described a robust and ongoing dialogue between their departments and the Department of Health:

‘… [*T*]hen it became a White Paper; at every stage there’s some consultation, even when it was a Bill, there have been some consultations. So for people to say, no, we haven’t been consulted, is really being disingenuous.’ (R2, Male, Ekhuruleni)

### Top-down and detached decision-making

The FPs described a top-down and detached governing structure where key stakeholders were not involved in the formulation of NHI policy and plans for implementation. Consequently, they expressed concerns that the bills would fail to achieve the buy-in from all role-players:

‘Ja, and I wonder if they’ve sold it well enough, because I think there’s a lot of resistance, as far as I can pick up, to the change. I think the private GPs are up in arms and scared of changing practices. I think the specialists are also worried, because the whole structure of services changes.’ (R8, Female, Metropolitan)

Family physicians described an underappreciation of their roles and described a sense of disempowerment to realise their potential for health systems strengthening, particularly within their own districts:

‘The unfortunate thing, only one hindrance that we have, is probably district management is not very supportive, all these things; they don’t want to see the importance of family medicine.’ (R5, Female, Ekhuruleni)

## Challenges to the successful implementation of National Health Insurance

### Uncertainty on the way forward

Family physicians were generally split on their opinions of how best the NHI should be implemented. Some held that NHI should be implemented in a reflexive manner, with cyclical implementation informed by experiential processes, while others felt that all necessary measures should be in place before NHI is rolled out:

‘I don’t know. To be able to understand if, really, it’s worth it, you have to start using it and exploring it, you have to become, yourself, involved in that, so you can say, yes, it works or it doesn’t work.’ (R4, Female, Metropolitan)

### Support from other governmental structures

Concerns around NHI implementation extended beyond healthcare, often indicating a non-coordination between governmental sectors. For example, FPs held that higher education (and the training of medical practitioners) was outdated, favouring a centralised model of healthcare – inconsistent with the focus on PHC inherent in the NHI bill. In addition, sectors such as home affairs failed to regulate migration into South Africa or provide a registry of citizens for access to care. Family physicians held that these other systems (governmental structures) would also need reform to aid the facilitation of NHI strategies:

‘[*T*]he issues that have destabilised the health care system that require attention, are really human resources, the financial management, the procurement and supply chain management, and the maintenance of infrastructure and equipment.’ (R8, Female, Metropolitan)

‘[*A*] lot of the universities, is very specialist-orientated, and the emphasis on primary care, general practitioners, district services, I think, is not yet well into the curriculums.’ (R6, Male, Tshwane)

‘And here, we’re talking … I’m not against the foreigners, I’m a foreigner myself … but I’m contributing taxes, and we know how many of the foreigners are not contributing taxes to this country.’ ‘… they’re not interested how they’re going to pay, because some of them, they don’t even have money to pay.’ (R4, Female, Ekhuruleni)

### Background detractors

Several FPs made references to ‘hidden’ role-players within the private sector who stood to lose if NHI was implemented, actively impeding implementation processes:

‘I don’t think it’s a big story, I think people are making it a big story, because of interests. I mean, if only 28% of the money available in private, is being paid to frontline guys, plus specialists, where is the remaining 70% of money? … and they sway a lot of power, they hold a lot of power, and so that needs to be broken, that hold needs to be broken.’ (R10, Male, Sedibeng)

### Corruption and poor governance

Some FPs described how previous engagements negatively shaped their views on NHI and the governing party’s true intentions. Emotive descriptions portrayed a sense of disillusionment and anger from FPs, who had taken the lead on NHI, towards the DoH:

‘And now, I understand it, is that there’s more focus on who’s going to get what bit of the pie in the corruption, than actually what needs to be implemented … The experience is that state owned entities, and programmes and systems, are designed so that corruption can be tolerated. … I say this, after years of putting my soul into it. I’ve worked very hard in terms of concepts, in terms of advocacy, in terms of detailed proposals. I developed a detailed document on a district-based system for managing the NHI, in terms of health catchment areas, with detailed description of an integrated, coordinated system … we spent months, nights, to prepare a tender document among six universities … it cost us millions, and the Department of Health blatantly reneged [*went back*] on their agreement.’ (R9, Male, Tshwane)

These experiences detracted from the confidence in the current governing parties to implement NHI:

‘No! Be frank and honest. The SOEs [State-owned enterprises] are just swallowing all the state resources; what for? Who actually uses SAA [*South African Airways*]? Hm? Tell me? Any poor passenger using SAA? No! So why are you pumping money there?’ (R1, Male, Metropolitan)

Cynicism seemed to reach a boiling point as some FPs called for regime change:

‘… [*T*]he biggest problem I see, is the people who are in position of power now … they are there to stay until they die.’ (R1, Male, Metropolitan)

‘The ethics in the leadership in South Africa is selfishness and greed. There’s no ethic, there’s no ideology; what is put forward is a farce, and it plays out. There’s a tolerance for corruption, there’s a tolerance for incompetence, and if you have enough incompetence, it creates the environment for corruption; and if you have enough corruption and incompetence, you have expensive chaos, or an expensive vacuum. It’s like eating your own children.’ (R9, Male, Tshwane)

## Suggestions for success

### A grassroots approach

A recurring suggestion was that of a robust engagement with all stakeholders. Emphasis was placed on a bottom-up approach. The basis for this assertion was seemingly two-fold: widespread consensus orientation on NHI policy, and the development of implementation strategies informed by the needs of particular districts:

‘I would start from the communities themselves, the populous, the people whom this thing is supposed to provide health care service to …’ (R1, Male, Metropolitan)

### Bolstered primary health care services

The opinions of FPs echoed several established tenets of UHC implementation, including a strong focus on reorienting the healthcare service delivery model to bolster PHC. This included the digitisation of healthcare records and integrated geographic information systems to map out communities and their respective needs:

‘I think every person in South Africa should have a Smart ID card … you drive into a place, you flash your card, it gets recorded electronically, and you’ve got all the information … not only a medical situation, but also the population-based information.’ (R6, Male, Tshwane)

Similarly, FPs held strong views on the role of decentralising healthcare authority to the districts, coupled with the need to capacitate districts to function independently:

‘They will be able to manage their own issues, in terms of they will get the funding, and spend it in an accountable and responsible way, or transparent way. But they are going to have some form of power to know what to spend on and not; they will be accountable … will be able to organise themselves …’ (R2, Male, Ekhuruleni)

### General practitioners as the backbone of primary health care

The role of general practitioners (GPs) in NHI was commonly mentioned. Family physicians seemed to view GPs as a pillar for NHI success. Getting the buy-in of GPs was regarded as a prerequisite to facilitate the merger of the public and private sectors, pivotal to strengthening PHC in South Africa. However, FPs that current GPs practice should be standardised to ensure best practice:

‘And it’s good, I think the future looks good, as far as I’m concerned; and the introduction of training, in terms of family physicians, is one thing we also need to really accelerate. So many GPs, their skills level are not up to the standard of NHI, or at least that’s what we think; but I don’t think they are at that level yet, so we need to demand a certain level or standard.’ (R2, Male, Ekhuruleni)

### Family physicians as champions of primary care

Family physicians held strong views on their role to play the implementation of NHI. Their frequent use of possessive pronouns implied a sense of ownership of PHC services and the need to drive UHC implementation:

‘… [*B*]ut on this issue of NHI, the politicians need to allow us to drive it … I think if you ask us to say, look, understand, this is what we want … give it to us what we need, then we start transforming … We are checking them off, and we can tick them, to say, Dr […] is already NHI compliant, and is fully ready for NHI … All those indices, we have indices, we have them, we mark them, we say, no, we are ready.’ (R7, Male, Sedibeng)

## Discussion

In this study we sought to explore the views and perceived engagement of FPs in NHI roll out in South Africa. Family physicians are primary care specialists with robust training in clinical governance and health system strengthening. These characteristics make the FP unique in their ability to drive PHC strengthening – a cornerstone of UHC success. As South Africa continues its phased implementation of NHI, the engagement and ownership of devolved governance and healthcare service delivery to the district health teams and FPs is critical in translating the ideology of NHI policy into strategic action.

### Views on National Health Insurance

The participants in our study expressed strongly supportive views on the conceptual framework of NHI. The FPs held that the current bipartite system of healthcare was inequitable and unsustainable. Furthermore, they felt that the principles of NHI, as a scheme that would see widespread investment in achieving UHC in South Africa, was a moral obligation to the citizens of South Africa. However, participants felt that the development of the NHI policy was conducted in a detached manner that failed to engage key stakeholders in South Africa’s healthcare system, most notably, the users of these healthcare services. The same sentiment has been found in other studies of healthcare workers (HCWs) in Ugu district in KwaZulu-Natal, Tshwane District, and Gauteng.^21,22^ Private GPs supported the idea of NHI as a means to address discrepancies in resource distribution, cost of private care, and improve the quality of public healthcare, but felt that government’s approach to them was ‘antagonistic’.

Despite the apparent support for the principles of UHC, the FPs in our study enumerated several concerns around the rollout of NHI. A prominent concern was that of the NHI policies – perceived as focusing only on financing structures with minimal consideration of the operational strategies to bolster PHC. Private GPs have also shown apprehension towards NHI because of the lack of clarity around NHI’s roll out and control of the risks.^24^ Similarly, ambivalence towards NHI was described among the public sector nursing manager in eThekwini because of a lack of clarity and uncertainty around the control of risks.^30^

Another commonly cited concern was that of wanton corruption. This concern was often tied to either perceived fraudulence and misconduct in other government sectors or personal experiences (and a sense of disillusionment among FPs) when working with the government on NHI pilot initiatives. The disillusionment described by the FPs (at the policy and planning level) in our study was found amongst various cadres of HCWs (including general practitioners,^20,23,24^ specialists,^17^ and professional nurses).^18,31^ For example, GPs in Gauteng who had been contracted to work at NHI pilot sites and seen government fail to compensate them. Additionally, and perhaps surprisingly, an attitude of uncertainty towards government was found among district managers^25^ in Gauteng province – the designated public sector managers responsible for the implementation of NHI.

### Communication and engagement

Communication on NHI (what it is and how it will be rolled out) was seen as inadequate, vague, and ambiguous. The respondents who reported that they had some clarity on the government’s plans for NHI had either actively researched NHI or made inferences based on how UHC was implemented in other settings. These findings were similar to those among doctors and professional nurses in the Eastern Cape where, while nearly 90% demonstrated an awareness of NHI, only 30% had received formal communication from government on the matter. The authors highlighted the importance of the media in driving awareness around NHI objectives and fostering a social cohesion towards the successful implementation of NHI as seen in Nigeria.^32^ The use of social media could prove influential in raising understanding among other cohorts where a lack of awareness has been described such as private care nursing staff,^18^ and public sector managers.^25^

General practitioners, considered the majority of primary care clinicians and central to phases I and II of implementation, also felt that they had little engagement from government.^20,23^ They described a willingness to engage and implement strategies; but described a dearth of detail around government implementation strategies.^20^ One group of GPs in the Western Cape expressed concerns around an antagonistic relationship between the private sector and the government^23^ – a group whose collaboration in NHI implementation of NHI was considered by the FPs in our study as critical to its success.

The lack of clarity around NHI contributes to the ongoing uncertainty amongst the users of the healthcare system, as well as civil society groups, namely the Helen Suzman Foundation, Section 27, and People’s Health Movement.

### Challenges to successful implementation

When considering challenges to the successful implementation of NHI, staffing, infrastructure within the public sector was seen as a major obstacle that would need to be addressed. The FPs felt that across all cadres of human resources there was a significant shortage of manpower. This sentiment has been a prominent concern in several other studies in KZN (general practitioners and nurses),^33^ The Eastern Cape (GPs and nurses),^19,20^ the Western Cape (GPs),^23^ Limpopo (GPs and nurses),^31^ as well as Gauteng (GPs, nurses, and managers).^18,22,25^ Further, in both our study and these studies, there was a sentiment that the human resources shortage would not be addressed in the near future.

A second major concern found in our study was that of inadequate infrastructure. Family physicians felt that the current public health sector infrastructure has not been maintained and has not grown to accommodate the increasing population size. Only two FPs in our study felt that facilities were ready to implement NHI. This finding was also found in another study where only 40% of HCWs at a tertiary facility considered their facility ready to implement NHI.

With regard to the employment of FPs, our study found that FPs experienced difficulty in motivating their respective human resources departments for the employment of FPs within their districts. A potential explanation for this is an ambivalence towards the value of FPs in the district managers,^25^ as well as the perceived (among some FP’s) appointment of incompetent health managers who were appointed on the basis of political affiliations, rather than competence.^22,23^

When considering the current health system structure, a recent study found that the perception of district managers mirrored that of the FPs, particularly with concerns around high levels of corruption within the Department of Health structures. Corruption has been described as common throughout the public sector in South Africa.^34^ A scoping review of UHC implementation in Ghana showed that corruption undermined the implementation process and would need to be closely monitored to prevent the same consequences in South Africa.^35^

An interesting finding of our study was the mention of hidden role players, predominantly within the private sector, that would purportedly impede the success of NHI. Participants highlighted the fact that the introduction of a regulated financing scheme would reduce the profits that private groups would normally enjoy (through inequitable profit sharing and third-party payoffs). Family physicians argued that these groups would actively antagonise any intervention (whether through misinformation, appeals, or otherwise) that could threaten their private wealth. The most recent health market inquiry,^36^ providing an overview of practices in the private sector, similarly found that large profit makers monopolised the health market and were not challenged by new entrants into the market.

A description of disillusionment was described by several FPs who had actively engaged the Department of Health in developing high-level NHI strategies and has not been described elsewhere. However, in NHI pilot sites, GPs have described failures to receive reimbursement from the government for their contractual work in the public sector. Other GPs have also described non-NHI related delays in compensation of up to 4 years when dealing with the workers compensation act.

### Suggestions for success

When considering what it would take for NHI to work in South Africa, FPs frequently cited a need for bolstering primary care, decentralising healthcare governance, eradicating corruption, and fostering open dialogue around NHI and its implementation. These tenants align well with the framework for action when implementing UHC in African countries.^37^ It is important to observe that these recommendations are only a framework and each country must adapt according to their local context. Universal Health Coverage implementation in African countries such as Nigeria and Ghana has demonstrated the importance of adhering to this framework – both countries have had shortcomings in effective decentralisation of healthcare as well as clinical governance and societal engagement. Systematic reviews, from the Asia Pacific^38,39^ as well as Latin America^8^ have conclusively demonstrated that even countries with a low GDP per capita can succeed when these frameworks are adhered to. The role of the FP would include advocacy for sustained investment in PHC strengthening and a shift in healthcare service orientation towards preventive and promotive medicine, while tailoring services to community needs. Additionally, an effective UHC would require multisectoral engagement to facilitate efficiencies and effectiveness of the health system such as electronic health records and population mapping.

In our study, FPs viewed themselves as instrumental in ensuring the successful implementation of UHC through the operationalisation of the district health team, community-oriented primary care strategies, and the gatekeeping of referrals. This opinion has been supported by studies on DHTs and their view on the role of the FP in the DHT.^10^ Despite this, FPs felt that their value proposition for PHC strengthening was underappreciated resulting in their exclusion from policy documents^13^ as well as human resource planning strategies.^17^ Family physicians held that their inclusion as primary care champions with a granular awareness of the operations at the primary care level would lead to contextual interventions with clear objectives and outcomes that could be measurable and monitored. This highlights the integrated training programme of the discipline of family medicine, as well as its utility in both primary and district healthcare service strengthening.

Views from FPs on infrastructure and human resources were split. While some FPs felt that infrastructure should be expanded and more staff employed, others felt that there should rather be a focus on the transformation of the current health system to better utilise the existing resources. A qualitative study conducted on NHI pilot studies using the theory of change, supported this viewpoint, highlighting the fact that less focus should be on money influx and more on the elements of the framework for actioning UHC.^6^

### Conclusion and recommendations

This study demonstrates that FPs are strongly supportive of the principles of UHC in seeking to improve the quality, coverage, and access to healthcare services for all citizens. Public healthcare services will require improved infrastructure and human resources for NHI roll out. Translation of the ideology found in the NHI bill into practical applications is required to garner trust, as well as actions, among stakeholders. Widespread investment in engaging stakeholders, including FPs, in policy formulation and strategic planning is required to foster consensus orientation and creating a sense of transparency among groups who describe several concerns around corruption in the public sector. The government should employ multiple channels, including social media, for this purpose.

Increased posts for FPs, as the champions of primary care, will create a body of NHI-supporters willing to drive the implementation process. Alongside this, healthcare governance should be devolved from national and provincial structures to the district levels, with the management structures that have been designed to support healthcare governance.

### Limitations

This study was conducted in a single district and is neither reflective of other district systems nor the FPs working there. Interviews were conducted online, in the early phases of the COVID-19 pandemic where the use of online platforms for communication was only starting to enjoy widespread uptake. This possibly hindered non-verbal communication and interview techniques familiar to both the researchers and respondents. Snowball sampling introduced community bias and may have resulted in a skewed sampling process, failing to reflect all.

Family physicians were from within the districts. Lastly, as all researchers were FPs, interviewing FPs, it is highly likely that social desirability bias could have shaped the responses within the interview.
